# Synthesis and characterisation of the ternary intermetalloid clusters {M@[As_8_(ZnMes)_4_]}^3−^ (M = Nb, Ta) from binary [M@As_8_]^3−^ precursors†

**DOI:** 10.1039/d2sc01748b

**Published:** 2022-05-16

**Authors:** Wei-Qiang Zhang, Harry W. T. Morgan, John E. McGrady, Zhong-Ming Sun

**Affiliations:** State Key Laboratory of Elemento-Organic Chemistry, Tianjin Key Lab of Rare Earth Materials and Applications, School of Materials Science and Engineering, Nankai University Tianjin 300350 China sunlab@nankai.edu.cn; Department of Chemistry, University of Oxford South Parks Road Oxford OX1 3QR UK john.mcgrady@chem.ox.ac.uk

## Abstract

The development of rational synthetic routes to inorganic arsenide compounds is an important goal because these materials are finding applications in many areas of materials science. In this paper, we show that the binary crown clusters [M@As_8_]^3−^ (M = Nb, Ta) can be used as synthetic precursors which, when combined with ZnMes_2_, generate ternary intermetalloid clusters with 12-vertex cages, {M@[As_8_(ZnMes)_4_]}^3−^ (M = Nb, Ta). Structural studies are complemented by mass spectrometry and an analysis of the electronic structure using DFT. The synthesis of these clusters presents new opportunities for the construction of As-based nanomaterials.

## Abstract 2

Two ternary intermetalloid clusters were constructed through binary intermetalloid clusters with a low valent group 12 metal salt. These clusters represent the first example of the structural transformation for intermetalloid clusters.

## Graphical abstract

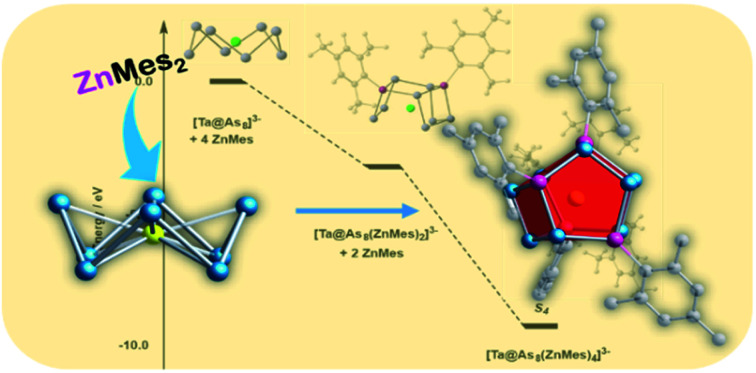

## Introduction

**Scheme 1:**
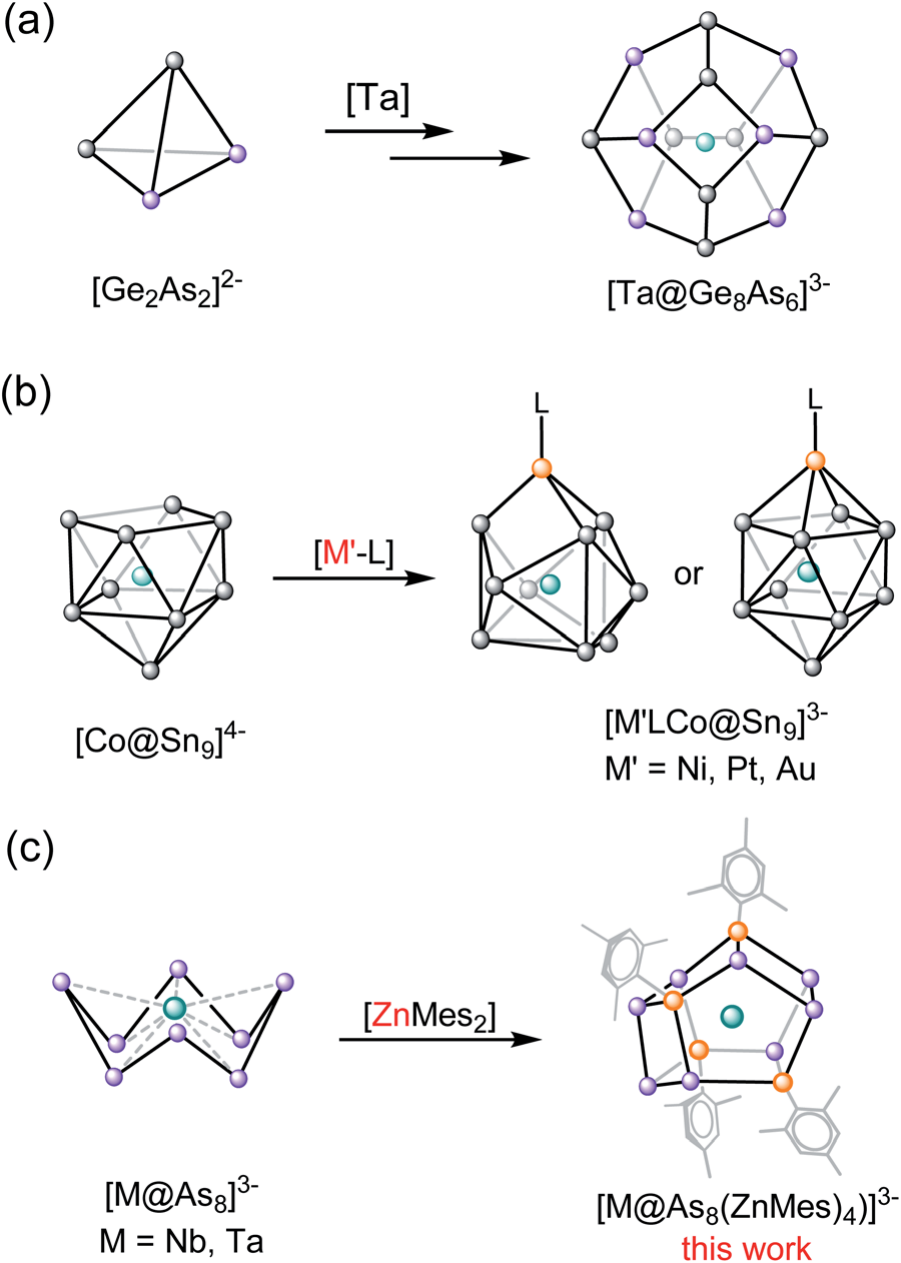
(a and b) Selected examples of ternary intermetalloid cluster anions of the general formula [M_*m*_@E_*x*_^1^E_*y*_^2^]^*q*−^ synthesised by different methods. (c) This work.

Ternary intermetalloid clusters have attracted a great deal of attention in the recent literature due to their complex electronic structures and also their potential role in materials science.^1–3^ The majority of known clusters in this class are synthesised either by extraction of the corresponding quaternary intermetallic phases or by the reaction of binary Tt/Pn or Tr/Pn Zintl anions with sources of low-valent transition metals, lanthanides or actinides.^4–6^Scheme 1a illustrates the extraction of the quaternary solid-state Zintl phase K/Ge/As/M (M = V, Nb, Ta) in ethylenediamine (en) in the presence of the sequestering agent, [2.2.2]crypt, which allows for the crystallization of cluster anions including 12-vertex [M@Ge_8_As_4_]^3−^ (M = V, Ta), and 14-vertex [M@Ge_8_As_6_]^3−^ (M = Nb, Ta).^7,8^ Clusters such as [Zn@Zn_5_Sn_3_Bi_3_@Bi_5_]^4−^,^9^ [Ni_2_@Sn_7_Bi_5_]^3−^,^10^ [Pd_3_@Sn_8_Bi_6_]^4−^,^11^ [Pd@Pd_2_Pb_10_Bi_6_]^4−^,^12^ and [Eu@Sn_6_Bi_8_]^4−^,^13^ have, in contrast, been accessed by reacting a salt of a binary mixed main group Zintl compound, [K([2.2.2]crypt)]_2_[TrBi_3_]·en (Tr = Ga, In, Tl) or [K([2.2.2]crypt)]_2_[Tt_2_Pn_2_]·en (Tt/Pn = Sn/Sb, Sn/Bi, Pb/Bi)^14,15^ with various organometallic compounds. A variant on this approach is to use a preformed binary intermetalloid where the central transition metal is already in place, surrounded by a shell of main-group atoms, as a starting material to react with other transition metal salts (Scheme 1b). This protocol has been used, for example, in the synthesis of [(L)MCo@Sn_9_]^3−^ from the reaction of [Co@Sn_9_]^4−^, extracted from the ternary phase “K_5_Co_3_Sn_9_”, with group 10-containing precursors such as [Ni(CO)_2_(PPh_3_)_2_], [Ni(cod)_2_], [Pt(PPh_3_)_4_] or [Au(PPh_3_)Ph].^16^ It is significant that these reactions involve only a simple ligand exchange reaction, and do not cause a substantial rearrangement of the binary inter-metalloid cluster core.^17^

The extension of this protocol to clusters containing group 15 elements has not been explored in detail, but there is a readily accessible family of starting materials available in the form of the crown-like [M@As_8_]^3−^ clusters, the first example of which, [Nb@As_8_]^3−^, was reported in 1986.^18^ Since then, the family has been extended to include [Mo@As_8_]^2−^,^19^ and [Cr@As_8_]^3−^,^20^ as well as the Sb analogues, [Mo@Sb_8_]^3−^ and[Nb@Sb_8_]^3−^.^20,21^ A broad survey of the electronic structure of the [M@Pn_8_]^*n*−^ family, encompassing M = Cr, Mo, Mn, Tc, Re, Ti, Zr, Hf and Pn = As, Sb; *n* = 1, 2, 3, has also been reported using density functional theory.^22^ The four As atoms on either side of the crown in the [M@As_8_]^*n*−^ unit form a region of high electron density, which has the potential to coordinate large alkali metals such as Rb, K, and hence form chain structures such as 
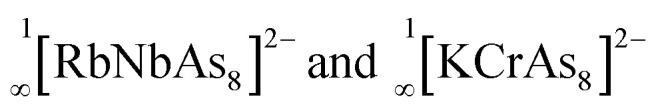
. In addition, the high oxidation state transition metal ion can serve as a 2-electron acceptor to form d^2^ complexes with Lewis basic reagents.^1*a*,22^ In a very recent study we showed that the reaction of [M@As_8_]^3−^, M = Nb, Ta, with a source of low-valent Cu can lead to the formation of an extended [As_16_]^10−^ ligand, where a tri-*cyclo* As_7_ unit is connected to a conserved As_8_ crown *via* a bridging As atom.^23^ Although mechanistic details are hard to elucidate, this reaction necessarily involves extensive cleavage and rearrangement of As–As bonds. In this paper we extend these studies to report the reactions of [K([2.2.2]crypt)]_3_[Nb@As_8_] or [K([2.2.2]crypt)]_2_[KTa@As_8_] with an organometallic source of Zn(ii), ZnMes_2_. In both cases the reactions generate 12-vertex cage type ternary intermetalloid clusters compounds, [K([2.2.2]crypt)]_3_{Nb@[As_8_(ZnMes)_4_]}·2en·tol (1) and [K([2.2.2]crypt)]_3_{Ta@[As_8_(ZnMes)_4_]}·en (2) where the As_8_^8−^ crown has been split into two As_4_^6−^ units, bridged by [ZnMes] fragments. The products are characterised by X-ray crystallography and mass spectrometry, and the electronic structures and formation pathways are explored using density functional theory.

## Results and discussion

**Fig. 1:**
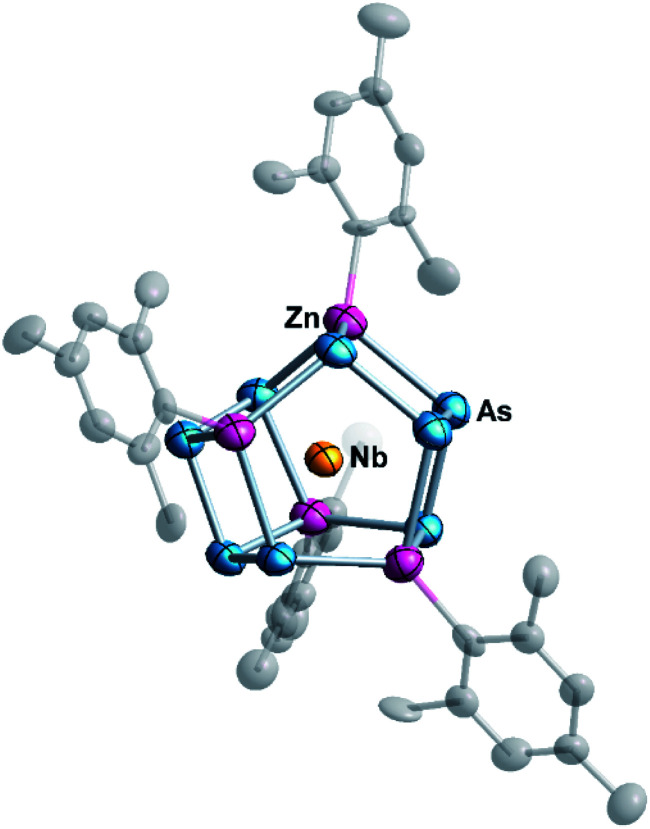
Molecular structure of the cluster {Nb@[As_8_(ZnMes)]_4_}^3−^ (1a) with thermal ellipsoids at the 50% probability level. The hydrogen atoms are omitted for clarity.

At elevated temperatures, ethylenediamine (en) solutions of [K([2.2.2]crypt)]_3_[Nb@As_8_] or [K([2.2.2]crypt)]_2_[KTa@As_8_] react with a toluene (tol) solution of ZnMes_2_ (Mes = 1,3,5-trimethylbenzene) to form [K([2.2.2]crypt)]_3_{Nb@[As_8_(ZnMes)_4_]}·2en·tol (1) or [K([2.2.2]crypt)]_3_{Ta@[As_8_(ZnMes)_4_]}·en (2), in *ca.* 30% and 25% yield, respectively. In the case of [K([2.2.2]crypt)]_2_[KTa@As_8_], one further equivalent of [2.2.2]crypt was added to the reaction to sequester the non-encrypted K^+^ ion. Diffusion of the reaction solution afforded dark-green block-like crystals of 1 and red-brown strip-like crystals of 2, both of which proved suitable for single-crystal X-ray diffraction study. Under similar reaction conditions, [K([2.2.2]crypt)]_3_{Ta@[As_8_(CdMes)_4_]} could also be isolated by replacing ZnMes_2_ with CdMes_2_ as starting regeant, but we have not yet been able to isolate crystals of sufficient quality to allow full structural characterization. Both 1 and 2 crystallise in the monoclinic space group *P*2_1_/*n* with a single {M@[As_8_(ZnMes)_4_]}^3−^ (M = Nb, Ta) anion and solvent molecules (en, tol) as well as three [K([2.2.2]crypt)]^+^ cations in the corresponding unit cell. Fig. 1 shows the molecular structure of the cluster anion in 1: full crystallographic details are given in ESI (Table S1†). For better visualization, different labels are used to distinguish the anionic cluster and the molecular compounds, and 1a–2a represent the anionic clusters in compounds 1–2, respectively.

The two anionic clusters {Nb@[As_8_(ZnMes)_4_]}^3−^ (1a) and {Ta@[As_8_(ZnMes)_4_]}^3−^ (2a) are isostructural and adopt almost perfect S_4_ point symmetry. They contain two tetrameric zig-zag [As_4_]^6−^ oligomers linked by four [ZnMes] groups in a μ:η^1:1:1:1^ coordination mode. The As–As bond lengths in 1a and 2a lie in the range 2.377–2.539 Å, values which are typical of As–As single bonds in polyarsenide Zintl clusters such as As_7_^3−^ and As_11_^3−^.^24^ The As–Zn distances (2.497(6)–2.693(6) Å for 1a, 2.500(9)–2.690(8) Å for 2a, respectively) are relatively widely dispersed because the As_4_^6−^ units contain As centres that are formally mono- and dianionic (in the centre and the termini of the As_4_ chains, respectively).^25^ For comparison, As–Zn bond lengths in [ZnAs_15_]^3−^ and [ZnAs_14_]^4−^ are at the shorter end of this range (2.488–2.536 Å,^26^ and 2.481–2.573 Å,^27^ respectively). The M–Zn (M = Nb, Ta) distances (2.778(9)–2.796(9) Å for 1a, 2.782(7)–2.795(8) Å for 2a) are considerably longer than those in organometallic compounds such as [(C_5_H_5_)_2_NbH_2_ZnC_5_H_5_] (2.541 Å)^28^ and [(CH_3_C_5_H_4_)_2_TaH(ZnC_5_H_5_)_2_] (2.589 Å)^29^ that are known to contain Nb/Ta–Zn bonds, precluding any direct metal–metal bonding in these clusters.

**Fig. 2:**
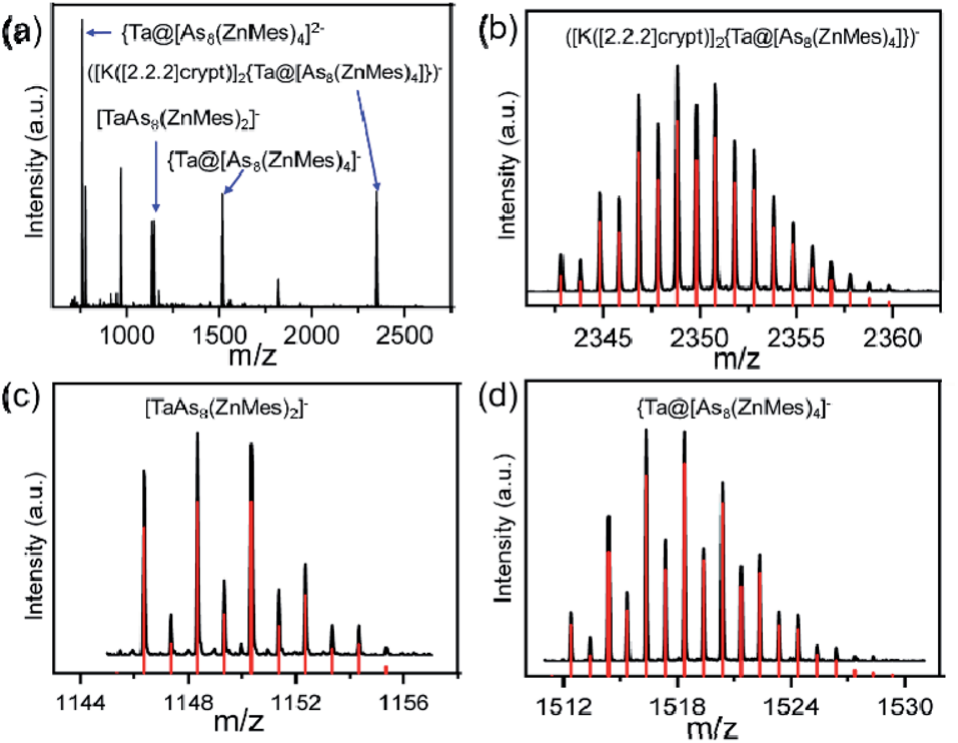
(a) Negative-ion ESI mass spectrum over the range of *m*/*z* 600–3000 of 2; (b–d) mass spectra of measured and calculated isotope patterns for peak ([K([2.2.2]crypt)]_2_{TaAs_8_(ZnMes)_4_})^−^ (*m*/*z* = 2348.8193), {TaAs_8_(ZnMes)_2_}^−^ (*m*/*z* = 1148.3488) and {TaAs_8_(ZnMes)_4_}^−^ (*m*/*z* = 1518.3754). The experimental mass distributions are depicted in black, and the theoretical masses of the isotope distribution are shown in red.

The overall stoichiometry of the reaction, [M@As_8_]^3−^ + 4 ‘ZnMes’ → [M@As_8_(ZnMes)_4_]^3−^, suggests a possible mechanistic pathway involving transient [Zn(i)Mes] fragments which cause the reductive cleavage of the As_8_^8−^ crown to form the two separate As_4_^6−^ fragments present in the product. Power and co-workers have reported a stable dimer of Zn(i)Ar (Ar = C_6_H_3_-2,6-[C_6_H_3_-2,6-(^i^Pr_2_)_2_]) produced by the reduction of ArZnCl with Na in ethanol,^30^ but there is no equivalent reducing agent present in these reactions, and the precise mechanism by which the low-valent Zn entities are formed remains uncertain. Reductive elimination of Mes_2_ from ZnMes_2_ is one possibility, and there is literature precedent for similar reactions,^31^ but we have not been able to confirm the presence of Mes_2_ in solution. The need for four equivalents of ZnMes to cleave two As–As bonds in the product suggests a stepwise pathway, and indeed we find evidence to support this in the negative-ion mass spectrum of 2 (Fig. 2), which shows prominent peaks for the parent ion at *m*/*z* = 1518.3754 ({TaAs_8_(ZnMes)_4_}^−^) and at *m*/*z* = 2348.8193 ([K([2.2.2]-crypt)]_2_{TaAs_8_(ZnMes)_4_})^−^, but also for the cluster with only two ZnMes units, {TaAs_8_(ZnMes)_2_}^−^ at *m*/*z* = 1148.3488. It is striking that all of the major peaks in the ESI-MS retain the 1 : 8 ratio of M to As, a clear indication of the robust nature of the [M@As_8_] unit, even under quite harsh reaction conditions.

**Fig. 3:**
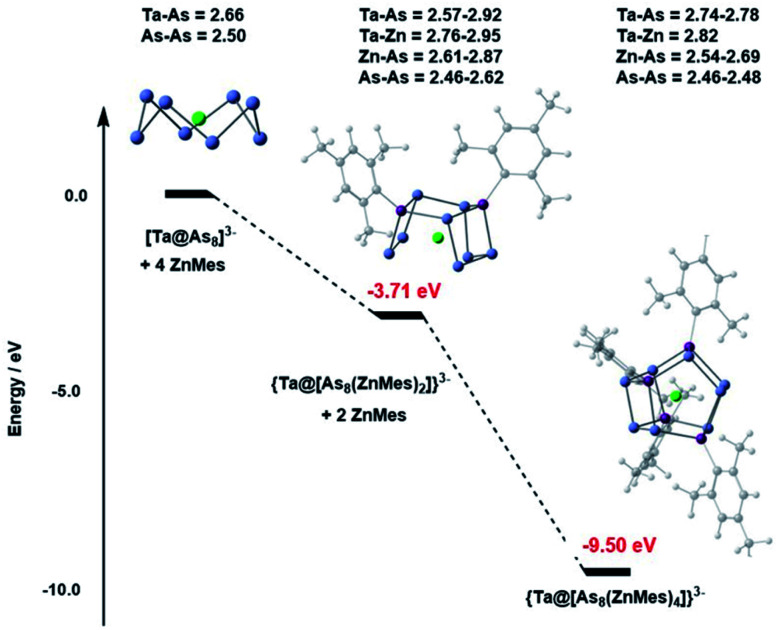
Potential energy profile for the cascade from [Ta@As_8_]^3−^ to {Ta@[As_8_(ZnMes)_2_]}^3−^ and then to {Ta@[As_8_(ZnMes)_4_]}^3−^ (2a). The profile for the corresponding reaction with Nb rather than Ta is very similar (relative energies are identical to within 0.05 eV).

The DFT-computed potential energy profile (ADF2021,^32^ PBE functional,^33^ triple-zeta polarised basis set – full details given in the ESI†) for the reaction of [Ta@As_8_]^3−^ with ZnMes shown in Fig. 3 reveals a cascade from reactants to products, with the {Ta@[As_8_(ZnMes)_2_]}^3−^ cluster (*E*_rel_ = −3.71 eV) clearly intermediate in energy between reactant (0) and product (−9.50 eV). The optimised structure of {Ta@[As_8_(ZnMes)_2_]}^3−^ confirms that only a single As–As bond has been broken in the intermediate, generating a contiguous As_8_ chain with a formal −10 charge. The computed energies therefore indicate that the stepwise addition of ZnMes to [Ta@As_8_]^3−^ leading to {Ta@[As_8_(ZnMes)_2_]}^3−^ and from there to {Ta@[As_8_(ZnMes)_4_]}^3−^ is a viable mechanism, at least thermodynamically, for cluster formation. We note, however, that the {Ta@[As_8_(ZnMes)_2_]}^3−^ intermediate is unstable with respect to disproportionation into reactants and products: 2[Ta@As_8_(ZnMes)_2_]^3−^ → [Ta@As_8_]^3−^ + [Ta@As_8_(ZnMes)_4_]^3−^, Δ*E* = −2.08 eV, so is unlikely to be amenable to isolation.

The cluster products obtained from the reaction with ZnMes_2_ make a striking contrast to the corresponding reactions of [M@As_8_]^3−^ with [CuMes(PPh_3_)_2_]·tol described in our previous paper,^23^ where two As_8_ units merged to form a single contiguous As_16_^10−^ ligand. The mechanistic details of the reactions with Cu are far from clear, not least because, unlike the reactions described here, the Nb/Ta : As ratio is different in reactants and products. Nevertheless, reductive cleavage of the As_8_ crown into As_4_ fragments is clearly not involved, and the key difference between the two metal reagents, ZnMes_2_ here and [CuMes(PPh_3_)_2_]·tol in the previous work, appears, therefore, to be the greater accessibility of transient monovalent “ZnMes” compared to zerovalent Cu.

### Comparison to other 12-vertex clusters

**Fig. 4:**
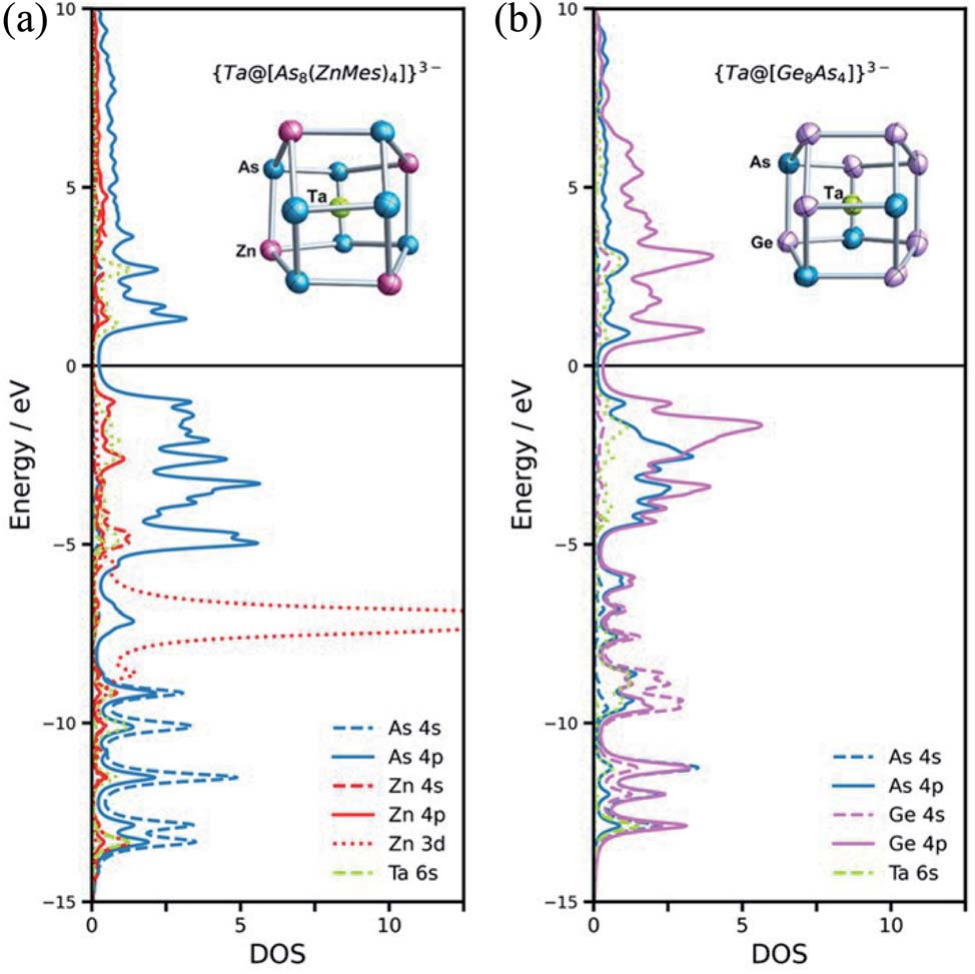
Structures and projected densities of states for the 12-vertex cluster anions {Ta@[As_8_(ZnMes)_4_]}^3−^ (2a) (a) and [Ta@Ge_8_As_4_]^3−^ (b), with thermal ellipsoids at the 50% probability level. The molecular structures of {Ta@[Ge_8_As_4_]}^3−^ is disordered, and the calculated minimum structure is presented here. The organic − Mes ligands are omitted in {Ta@[As_8_(ZnMes)_4_]}^3−^. The projected DOS is summed over all atoms of a given type.

A complementary perspective on the bonding in these clusters comes from noting their formal relationship to the {M@[Ge_8_As_4_]}^3−^ (M = V, Ta) clusters reported recently by Dehnen and co-workers (Fig. 4).^34^ The [ZnMes]^2−^ fragment is isolobal with Ge^−^ and also with As, and so the 60-electron, 12-vertex cages can be formulated as [Ge_8_As_4_]^8−^ in {Ta@[Ge_8_As_4_]}^3−^ and as [As_8_(ZnMes)_4_]^8−^ in {Ta@[As_8_(ZnMes)_4_]}^3−^. The projected densities of states (PDOS) for the two Ta-based clusters, {Ta@[As_8_(ZnMes)_4_]}^3−^ and [Ta@Ge_8_As_4_]^3−^, are also compared in Fig. 4. The contribution of Ta 5d orbitals to the occupied manifold is minor in both cases, suggesting that the Ta(v) oxidation state is a reasonable first approximation for the electronic structure. In the {Ta@[Ge_8_As_4_]}^3−^ case, there is substantial mixing between the Ge and As 4s/4p manifolds in both the occupied and virtual space, and the peaks that lie ∼1.0 eV above and below the Fermi level have Ge and As character in approximately equal proportion. In contrast, the As 4s/4p and Zn 4s/4p manifolds are well separated, and As 4p character dominates the window 5 eV either side of the Fermi level. Thus, whilst the structural chemistry and the formal valence electron count point to a close relationship between the two clusters, the bonding is significantly more ionic in the Zn/As clusters reported here than in the Ge/As analogues.

## Conclusions

In summary, we have established that the [Nb@As_8_]^3−^ and [Ta@As_8_]^3−^ anions can be used as synthetic precursors to ternary As-rich clusters where the M : As ratio of 1 : 8 is conserved. Their reactions with ZnMes_2_ yield cluster compounds of the general formula {M@[As_8_(ZnMes)_4_]}^3−^ (M = Nb, Ta), where two As–As bonds of the original crown-like As_8_^8−^ unit are cleaved to form two As_4_^6−^ fragments. The presence of clusters with two, rather than four, [ZnMes] units in the ESI-MS spectra suggests a step-wise pathway where [ZnMes] units are added sequentially with progressive fragmentation of the As_8_ ring. The reaction of [M@As_8_] units with low-valent transition metal organometallics described in this paper presents new possibilities for the construction of ternary arsenic-rich nanoclusters with tightly controlled stoichiometries.

## Data availability

Detailed experimental procedures, crystallographic supplementation, electrospray ionization mass spectrometry (ESI-MS) analysis, energy-dispersive X-ray (EDX) spectroscopic analysis, and quantum-chemical studies can be found in the ESI.†

## Author contributions

Z. M. S. conceived the project and designed the experiments. W. Q. Z. performed the synthesis and the single-crystal X-ray diffraction as well as analysed the data. H. W. T. M. and J. E. M. performed the quantum chemical calculations and analysed the data. All authors co-wrote the manuscript.

## Conflicts of interest

There are no conflicts to declare.

## Supplementary Material

SC-013-D2SC01748B-s001

SC-013-D2SC01748B-s002
