# Supplementary material for: Synthesis and characterisation of the ternary intermetalloid clusters {M@[As8(ZnMes)4]}3− (M = Nb, Ta) from binary [M@As8]3− precursors
Source: Chem Sci. 2022 May 16;13(22):6744–8. doi: 10.1039/d2sc01748b (PMC9172560; doi:10.1039/d2sc01748b)
Supplement: SC-013-D2SC01748B-s001 [file SC-013-D2SC01748B-s001.pdf]

Electronic Supplementary Material (ESI) for Chemical Science.
This journal is © The Royal Society of Chemistry 2022

Supporting Information

Synthesis and characterisation of the ternary intermetalloid clusters $\{M@[As_8(ZnMes)_4]\}^{3-}$ (M = Nb, Ta) from binary $[M@As_8]^{3-}$ precursors.

Wei-Qiang Zhang,[a] Harry W. T. Morgan[b], John E. McGrady[b]*, and Zhong-Ming Sun[a]*

State Key Laboratory of Elemento-Organic Chemistry, Tianjin Key Lab of Rare Earth Materials and Applications, School of Materials Science and Engineering, Nankai University Tianjin 300350 (China) E-mail: sunlab@nankai.edu.cn

Department of Chemistry, University of Oxford, South Parks Road, Oxford OX1 3QR, UK. E-mail: john.mcgrady@chem.ox.ac.uk

Table of Contents

1. Experimental Procedures....S2
2. Supplementary Crystallographic Information....S3
3. ESI-MS Studies....S7
4. Energy Dispersive X-ray (EDX) Spectroscopic Analysis....S9
5. Computational details....S10
References....S15

1. Experimental Procedures

All manipulations and reactions were performed under a dry nitrogen atmosphere in the glove box, as the clusters, in common with the majority of Zintl compounds, are sensitive to air and moisture. Ethylenediamine (en, Aldrich, 99%) and toluene (tol, Aldrich, 99.8%) were distilled over sodium metal and sodium/benzophenone, respectively, and were stored in gas-tight ampoules under nitrogen. [2.2.2]crypt (4,7,13,16,21,24-Hexaoxa-1,10-diazabicyclo (8.8.8) hexacosane, purchased from Sigma-Aldrich, 98%) was dried in vacuum for 12 h prior to use. The organometallic precursor of $ZnMes_2$ was synthesized according to the reported literature.[1] The precursors $[K([2.2.2]crypt)]_3[Nb@As_8]$ and $[K([2.2.2]crypt)]_2[KTa@As_8]$ were prepared according to the literature procedure.[2]

Synthesis of $[K([2.2.2]crypt)]_3\{Nb@[As_8(ZnMes)_4]\}\cdot 2en\cdot tol$ (1)

$[K([2.2.2]crypt)]_3[Nb@As_8]$ (50 mg, 0.025 mmol) was dissolved in 2 mL en and stirred for 10 min to yield red-brown solution. $ZnMes_2$ (30 mg, 0.10 mmol) was dissolved in 0.5 mL tol in a separate vial. The content of the second vial was added dropwise to the first vial. The reaction mixture was stirred for 2.5 h at 65 ℃ to yield a brown-green solution. The resulting solution was filtered through glass wool and transferred to a test tube, then carefully layered with toluene (3 mL) to allow for crystallization. After 1 month, dark-green block-like crystals of $[K([2.2.2]crypt)]_3\{Nb@[As_8(ZnMes)_4]\}\cdot 2en\cdot tol$ was observed in the bottom of the test tube. Yield: 21.7mg (30%) (based on the amount of $[K([2.2.2]crypt)]_3[Nb@As_8]$ used).

Synthesis of $[K([2.2.2]crypt)]_3\{Ta@[As_8(ZnMes)_4]\}\cdot en$ (2)

$[K([2.2.2]crypt)]_2[KTa@As_8]$ (50 mg, 0.03 mmol) and [2.2.2]crypt (15 mg, 0.03 mmol) were weighed into a 10 mL vial inside a glovebox and dissolved in 2 mL en solution and then the mixture was stirred for 10 min at room temperature to yield a red-brown solution. $ZnMes_2$ (30 mg, 0.10 mmol) was dissolved in 0.5 mL toluene (tol) in a separate vial. The content of the second vial was added dropwise to the first vial. The reaction mixture was stirred for 1.5 h at 65 ℃ to yield a brown solution. The resulting solution was filtered through glass wool and transferred to a test tube, then carefully layered with toluene (3 mL) to allow for crystallization. After 15 days, red-brown strip-like crystals of $[K([2.2.2]crypt)]_3\{Ta@[As_8(ZnMes)_4]\}\cdot en$ were observed in the bottom of the test tube. Yield: 21.1 mg (25%) (based on the amount of $[K([2.2.2]crypt)]_2[KTaAs_8]$ used).

Crystallographic methods

Suitable crystals from **1**-**2** were selected for X-ray diffraction analyses. Crystallographic data were collected on Rigaku XtalAB Pro MM007 DW diffractometer with graphite monochromated Cu Kα radiation (λ = 1.54184 Å). The structures of crystals **1**-**2** were solved using direct methods and then refined using SHELXL-2014 and Olex2.[3] All the non-hydrogen atoms were refined anisotropically, except for those in the split positions. In compound **1**, the solvent molecules have disordered structures, which were resolved using the mSplit process in Olex2. A summary of the crystallographic data for the title compounds were listed in Table S1, and selected bond distances was given in the Table S2-S3. CCDC entries 2078235 (**1**) and 2093790 (**2**) for compounds **1**-**2** contain the supplementary crystallographic data for this paper. These data can be obtained free of charge from the Cambridge Crystallographic Data Centre (www.ccdc.cam.ac.uk/data_requested/cif).

Electrospray Ionization Mass Spectrometry (ESI-MS) Investigations:

Negative ion mode ESI-MS of the MeCN solutions of crystals of **2** was measured on an LTQ linear ion trap spectrometer by Agilent Technologies ESI-TOF-MS (6230). The spray voltage was 5.48 kV and the capillary temperature was kept at 300 °C. The capillary voltage was 30 V. The samples were prepared inside a glovebox and very rapidly transferred to the spectrometer in an airtight syringe by direct infusion with a Harvard syringe pump at 0.2 mL/ min.

Energy Dispersive X-ray (EDX) analysis:

EDX analysis was performed to support the element composition that was proposed by the XRD experiment. These were carried out using a scanning electron microscope (Hitachi S-4800) equipped with a Bruker AXS XFlash detector 4010. Data acquisition was performed with an acceleration voltage of 15 kV and an accumulation time of 120 s.

Quantum chemical methods:

All calculations described in this paper were performed with the Amsterdam Modelling Suite (AMS) package of program, version AMS2021.104.[4] For all geometry optimizations, a triple-zeta quality basis set of Slater-type orbitals was used, supplemented by a single set of polarization functions. Electrons up to and including 1s (C), 2p (Zn), 3p (Ge, As), 3d (Nb) and 4d (Ta), were treated using the frozen core approximation.[5] All calculations use the gradient-corrected approximations to the exchange- correlation functional proposed by Perdew, Burke and Ernzerhof (PBE).[6] Scalar relativistic effects were included using the Zeroth order relativistic approximation (ZORA).[7] Vibrational frequencies were computed analytically, and all reported stationary points were confirmed to be minima through the absence of imaginary frequencies. The confining effect of cations in the solid state were simulated by using a continuum solvent model (COSMO) with a dielectric constant of ε_r = 100.[8] Density of states plots were generated by broadening the discrete energy levels using a Lorentzian with FWHM of 0.15 eV.

2. Supplementary Crystallographic Information

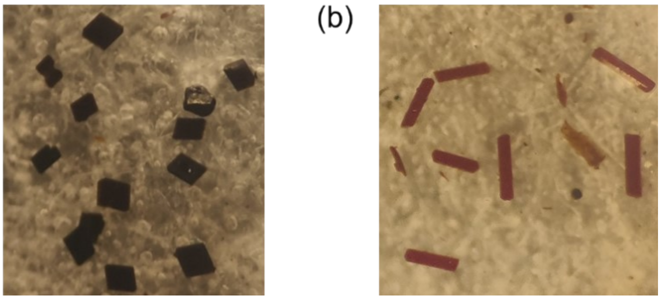

Figure S1. Crystals of [K([2.2.2]crypt)]$_3${Nb@[As$_8$(ZnMes)$_4$}·2en·tol (a) and [K([2.2.2]crypt)]$_3${Ta@[As$_8$(ZnMes)$_4$]}·en (b) dispersed in silicon oil, respectively.

Table S1. X-ray measurements and structure solution of **1**-**2**.

Compound	**1**	**2**
CCDC number	2078235	2093790
Empirical formula	$C_{101}H_{175}N_{10}O_{18}K_3NbZn_4As_8$	$C_{92}H_{160}As_8K_3N_8O_{18}TaZn_4$
Formula weight	2888.55	2825.36
Temperature/K	100.00(10)	100.00(10)
Crystal system	monoclinic	monoclinic
Space group	$P2_1/n$	$P2_1/n$
a/Å	16.9909(2)	17.0926(2)
b/Å	33.0617(4)	32.8161(4)
c/Å	22.6750(2)	22.5199(3)
α/°	90	90
β/°	104.5190(10)	104.2903(14)
γ/°	90	90
Volume/Å^3	12330.9(2)	12240.8(3)
Z	4	4
ρ_{calc}g/cm^3	1.556	1.533
μ/mm^{-1}	5.397	6.255
$F(000)$	5908	5704
2Θ range for data collection/°	7.48 to 133.986	7.584 to 133.996
Reflections collected	62745	63830
Data/restraints/parameters	21908/1/1326	21784/1/1219
Goodness-of-fit on F^2	1.097	1.068
Final R indexes [I>=2σ (I)]	R_1 = 0.0406, wR_2 = 0.1011	R_1 = 0.0472, wR_2 = 0.1204
Final R indexes [all data]	R_1 = 0.0452, wR_2 = 0.1031	R_1 = 0.0593, wR_2 = 0.1264
Largest diff. peak/hole / e Å^{-3}	0.99/-0.93	1.504/-1.844

^{a} $R_1 = \sum||F_o|-|F_c||/\sum|F_o|$; $wR_2 = \{\sum w[(F_o)^2-(F_c)^2]^2/\sum w[(F_o)^2]^2\}^{1/2}$
^{b} GooF = $\{\sum w[(Fo)^2-(Fc)^2]^2/(n-p)\}^{1/2}$

Table S2. Selected bond distances (Å) for the [K([2.2.2]crypt)]$_3${Nb@[As$_8$(ZnMes]$_4$}·2en·tol (**1**)

	Experimental		Experimental
Nb1-As1	2.728(5)	Nb1-As2	2.728(5)
Nb1-As3	2.719(5)	Nb1-As4	2.721(5)
Nb1-As5	2.728(5)	Nb1-As6	2.726(5)
Nb1-As7	2.722(5)	Nb1-As8	2.687(5)

Nb1-Zn1	2.793(5)	Nb1-Zn2	2.796(6)
Nb1-Zn3	2.793(5)	Nb1-Zn4	2.778(6)
As1-Zn1	2.497(6)	As1-Zn2	2.599(6)
As2-Zn4	2.693(6)	As3-Zn2	2.632(6)
As4-Zn3	2.530(6)	As4-Zn4	2.588(6)
As5-Zn3	2.614(6)	As5-Zn2	2.539(6)
As6-Zn1	2.661 (6)	As8-Zn1	2.625(6)
As7-Zn3	2.659(6)	As8-Zn4	2.503(6)
Zn1-C55	2.00(4)	Zn2-C64	2.01(4)
Zn3-C73	2.00(4)	Zn4-C82	2.00(4)
As1-As2	2.442(5)	As2-As3	2.419(5)
As3-As4	2.419(6)	As5-As6	2.423(6)
As6-As7	2.426(6)	As7-As8	2.427(6)

Table S3. Selected bond distances (Å) for the [K([2.2.2]crypt)]$_3${Ta@[As$_8$(ZnMes)$_4$]}·en (**2**)

	Experimental		Experimental
Ta1-As1	2.676(6)	Ta1-As2	2.729(6)
Ta1-As3	2.737(6)	Ta1-As4	2.713(6)
Ta1-As5	2.709(6)	Ta1-As6	2.727(6)
Ta1-As7	2.733(6)	Ta1-As8	2.712(6)
Ta1-Zn1	2.790(7)	Ta1-Zn2	2.797(7)
Ta1-Zn3	2.795(8)	Ta1-Zn4	2.782(7)
As1-Zn3	2.617(9)	As1-Zn4	2.504(9)
As2-Zn1	2.647(9)	As4-Zn2	2.537(8)
As3-Zn3	2.656(9)	As4-Zn1	2.608(9)
As5-Zn4	2.580(9)	As5-Zn1	2.529(9)
As6-Zn2	2.624(9)	As8-Zn2	2.595(9)
As7-Zn4	2.690(8)	As8-Zn3	2.500(9)
Zn1-C55	2.00(5)	Zn2-C64	2.01(5)
Zn3-C73	2.00(5)	Zn4-C82	2.00(5)
As1-As2	2.430(8)	As2-As3	2.427(8)
As3-As4	2.429(8)	As5-As6	2.420(8)

As6-As7	2.419(8)	As7-As8	2.448(8)

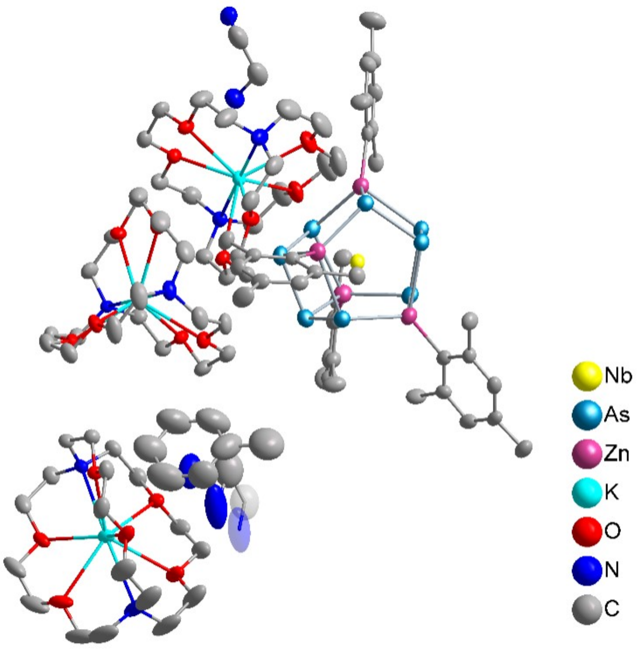

Figure S2. Asymmetric unit of **1** with the cluster fragment. Thermal ellipsoids are drawn at 50% probability. Positions of disordered atoms of solvent molecule are given in semitransparent mode. Hydrogen atoms have been omitted for clarity.

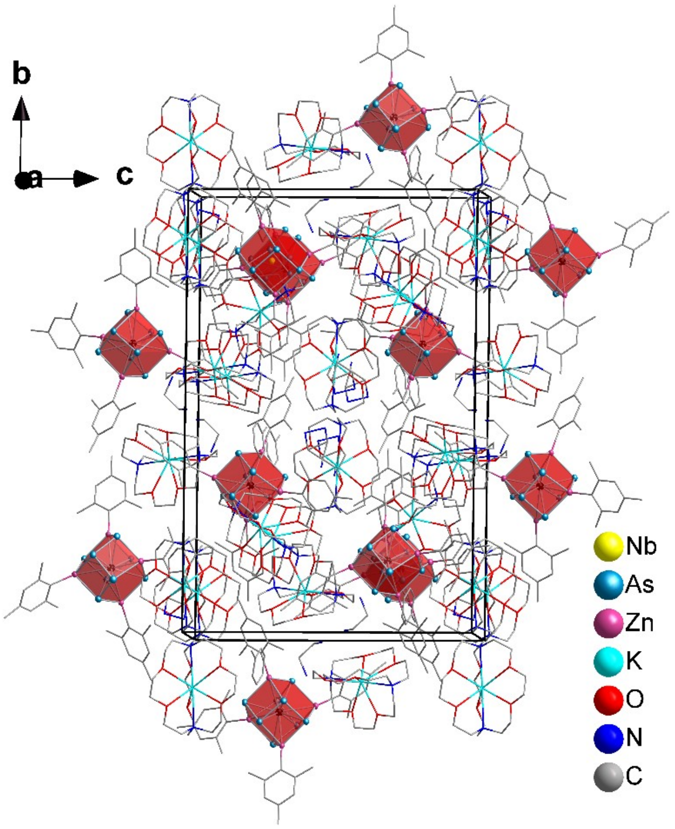

Figure S3. Packing of cations and anions in the structure of compound **1**. Anionic clusters are shown as polyhedral. Thermal ellipsoids are drawn at 50% probability. Hydrogen atoms have been omitted for clarity.

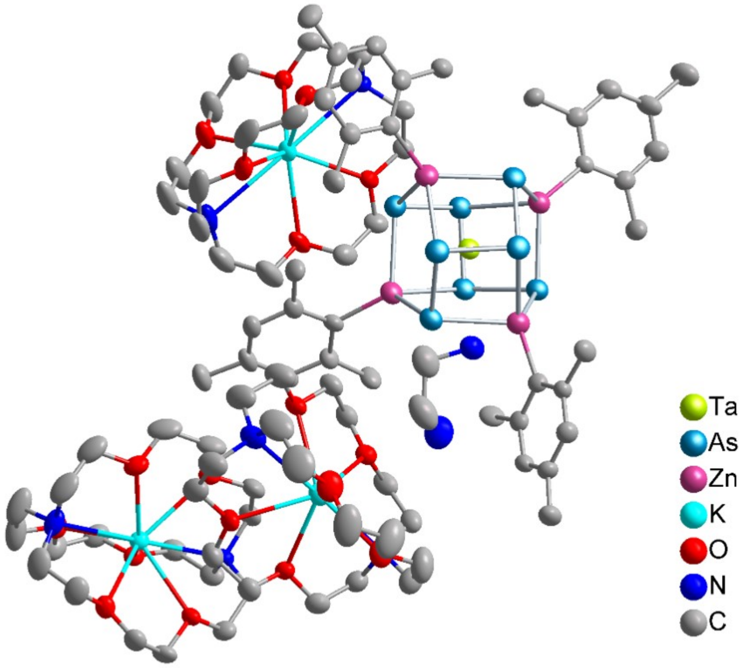

Figure S4. Asymmetric unit of **2** with the cluster fragment. Thermal ellipsoids are drawn at 50% probability. Hydrogen atoms have been omitted for clarity.

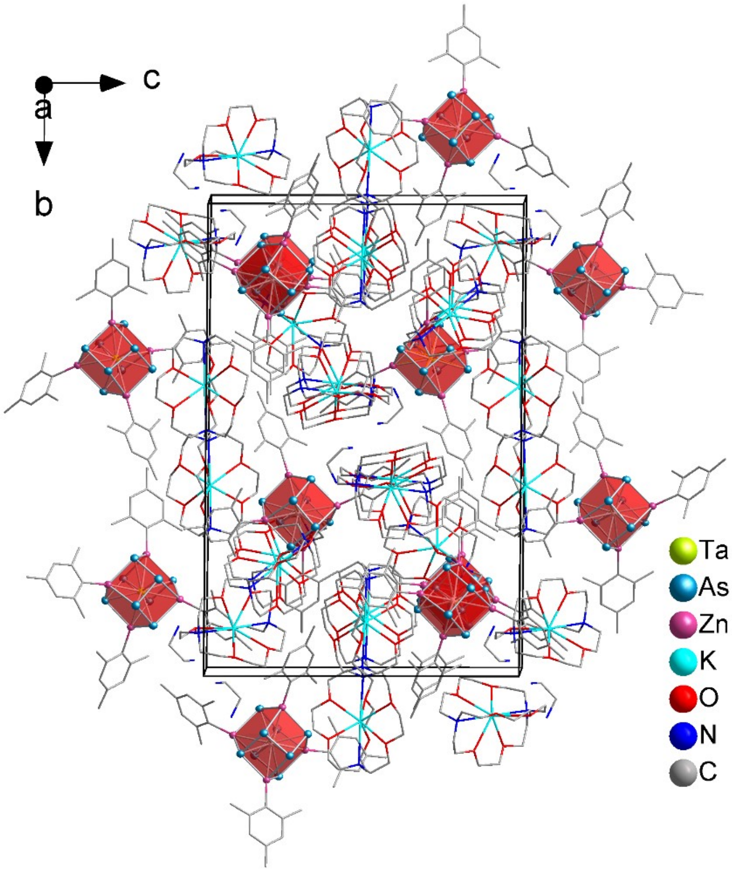

Figure S5. Packing of cations and anions in the structure of compound **2**. Thermal ellipsoids are drawn at 50% probability. Hydrogen atoms have been omitted for clarity.

3. ESI-MS Studies

The negative ion mode electrospray ionization (ESI) mass spectra of a fresh solution of single crystals of $[K([2.2.2]crypt)]_3\{Ta@[As_8(ZnMes)_4]\}\cdot en$ (**2**) in MeCN solution, indicating seven variations of the mass peak as measured (upper part of each panel) and simulated spectrum (lower part of each panel). Prominent amongst these are $\{[K([2.2.2]crypt)]_2\{Ta@[As_8(ZnMes)_4]\}\}^-$ (m/z = 2348.8193) and $\{Ta@[As_8(ZnMes)_4]\}^-$ (m/z = 1518.3754). Several other signals of cluster-containing peaks were shown in Figures S7-8.

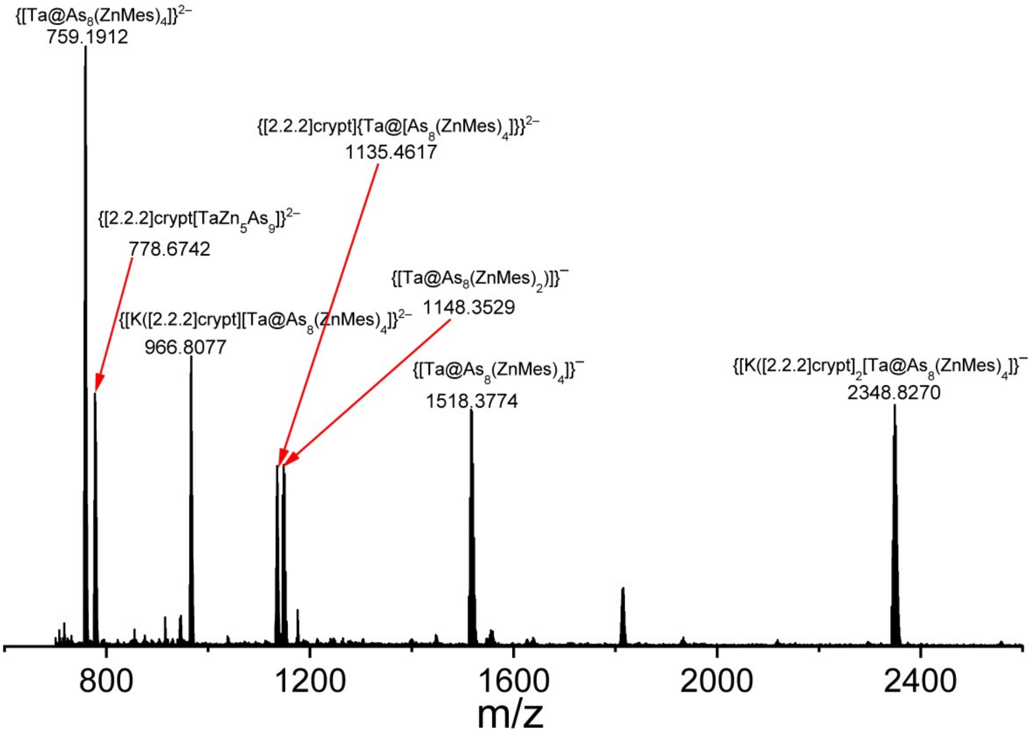

Figure S6. Overview ESI mass spectrum in negative ion mode of a freshly dissolved crystalline sample of $[K([2.2.2]crypt)]_3\{Ta@[As_8(ZnMes)_4]\}\cdot en$ in MeCN.

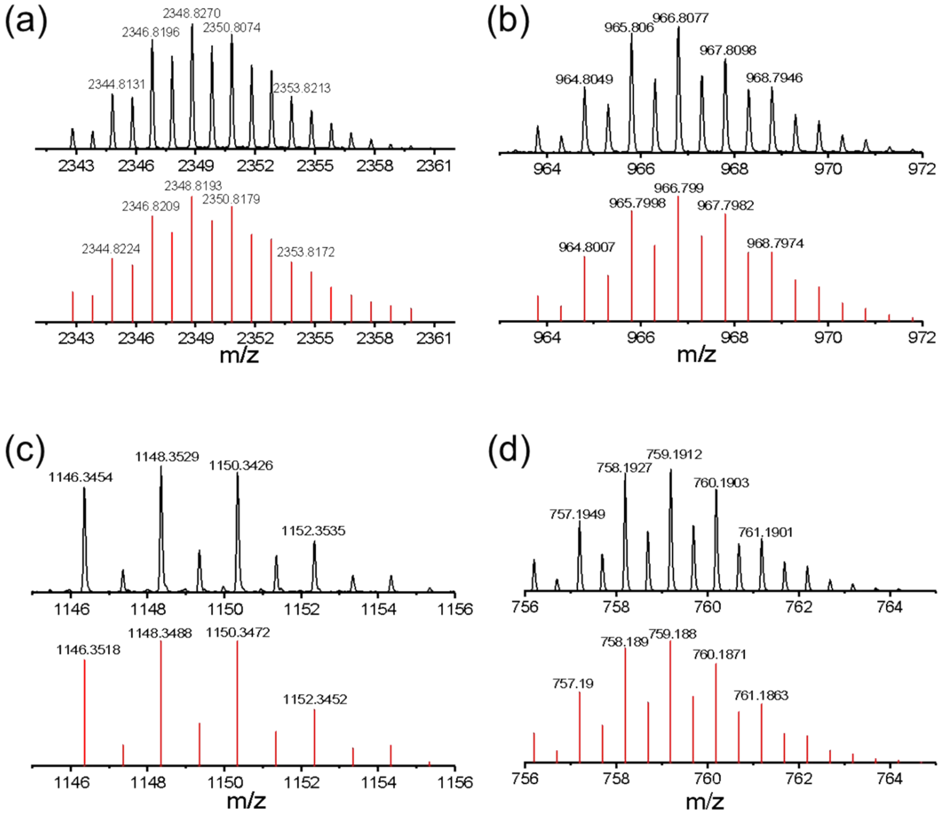

Figure S7. Measured (top) and simulated (bottom) spectrum of the fragment $\{[K([2.2.2]crypt]_2\{Ta@[As_8(ZnMes)_4]\})^-$ (a), $([K([2.2.2]crypt)]\{Ta@[As_8(ZnMes)_4]\})^{2-}$ (b), $[TaAs_8(ZnMes)_2]^-$ (c), $\{Ta@[As_8(ZnMes)_4]\}^{2-}$ (d).

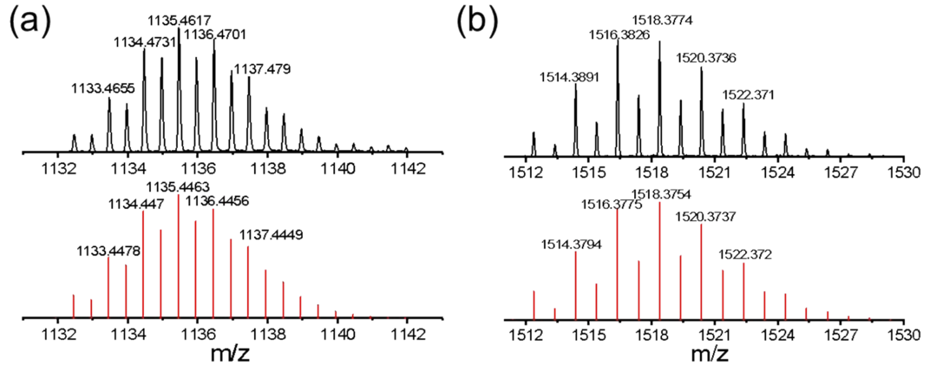

Figure S8. Measured (top, black) and simulated (bottom, red) spectrum of the fragment $\{([2.2.2]crypt)_2\}\{Ta@[As_8(ZnMes)_4]\}\}^{2-}$ (a), $\{Ta@[As_8(ZnMes)_4]\}^{-}$ (b).

4. Energy Dispersive X-ray (EDX) Spectroscopic Analysis

EDX analysis on **1**-**2** (Figure S9-10) was performed by a scanning electron microscope (FE-SEM, JEOL JSM-7800F, Japan). Data acquisition was performed with an acceleration voltage of 15 kV and an accumulation time of 120 s. Multiple crystals were used for EDX spectroscopic analysis, and data were recorded: different positions on one single crystal and different positions on other single crystals.

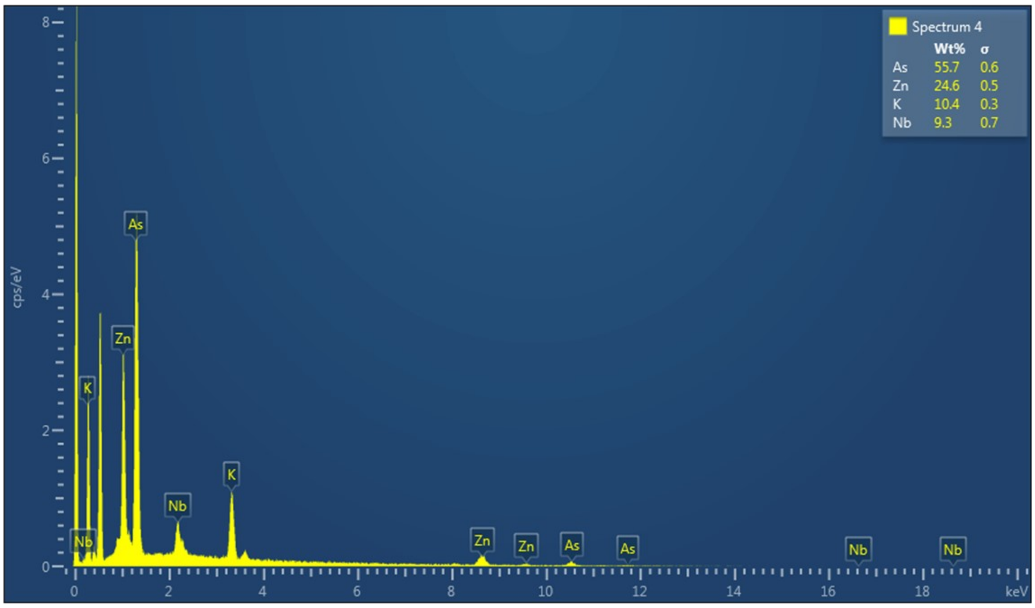

Figure S9. EDX analysis of [K([2.2.2]crypt)]$_3${Nb@[As$_8$(ZnMes)$_4$)]}·2en·tol (**1**).

Element	wt%	σ	Experimental / Calculated Atom %
K	10.4	0.3	17.91/18.75
Zn	24.6	0.5	25.3/25
As	55.7	0.6	50/50
Nb	9.3	0.7	6.7/6.25

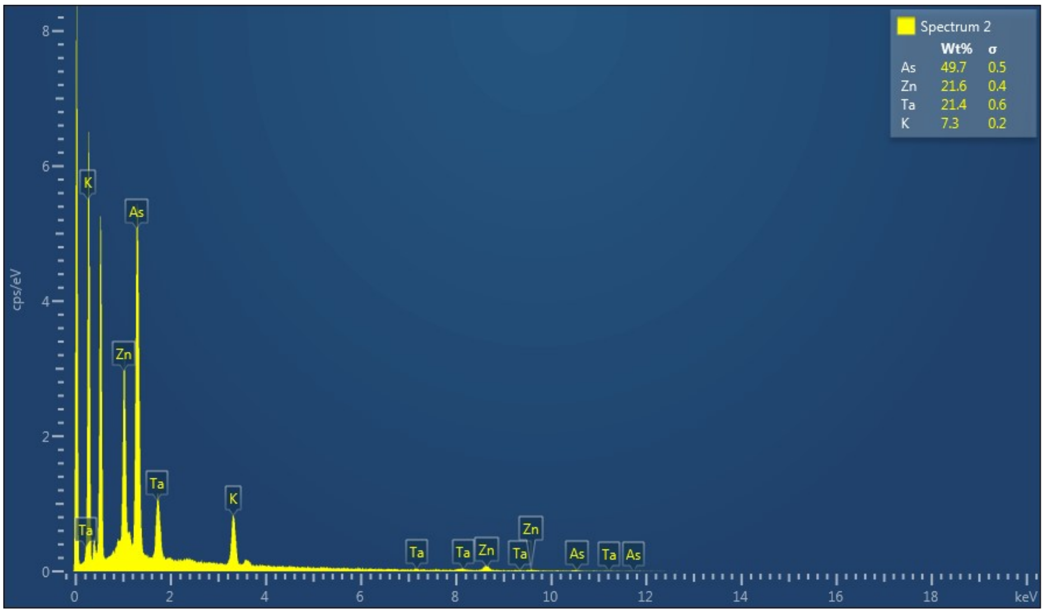

Figure S10. EDX analysis of [K([2.2.2]crypt)]$_3${Ta@[As$_8$(ZnMes)$_4$)]}·en (**2**).

Element	wt%	σ	Experimental / Calculated Atom %
K	7.3	0.2	14.38/18.75
Zn	21.6	0.4	25.4/25
As	49.7	0.5	51.1/50
Ta	21.4	0.6	9.1/6.25

5. Computational details

Table S4 Summary of total energies for all species computed using DFT (PBE functional with a TZP Slater-type basis, ZORA scalar relativistic approach and a relative permittivity of 100).

ZnMes	E = –120.190 eV
$[NbAs_8]^{3–}$	E = –56.582 eV
$[TaAs_8]^{3–}$	E = –55.933 eV
$[NbAs_8(ZnMes)_2]^{3–}$	E = –300.618 eV
$[TaAs_8(ZnMes)_2]^{3–}$	E = –300.023 eV
$[NbAs_8(ZnMes)_4]^{3–}$	E = –546.717 eV
$[TaAs_8(ZnMes)_4]^{3–}$	E = –546.193 eV

Table S5. Optimised cartesian coordinates (in Å) of all clusters.

ZnMes E = –120.190 eV

Zn	-2.218573	-0.407432	0.000000
C	-0.214048	-0.031644	0.000000
C	0.476676	0.087623	1.220889
C	0.476676	0.087623	-1.220889
C	1.861595	0.326260	1.203272
C	1.861595	0.326260	-1.203272
C	2.570471	0.449879	0.000000
H	2.400230	0.412913	2.152488
H	2.400230	0.412913	-2.152488
C	4.051774	0.733834	0.000000
H	4.539415	0.317493	0.892581
H	4.539415	0.317493	-0.892581
H	4.242432	1.819734	0.000000
C	-0.244060	-0.038295	-2.544114
H	0.446343	0.080210	-3.390448
H	-0.731360	-1.021481	-2.639416
H	-1.032513	0.724569	-2.640306
C	-0.244060	-0.038295	2.544114
H	0.446343	0.080210	3.390448
H	-1.032513	0.724569	2.640306
H	-0.731360	-1.021481	2.639416

$[Nb@As_8]^{3–}$ E = –56.582 eV

Nb	-0.000000	0.000000	0.000000
As	-0.977005	2.358700	-0.776085
As	-2.358700	0.977005	0.776085
As	2.358700	0.977005	-0.776085
As	0.977005	2.358700	0.776085
As	-2.358700	-0.977005	-0.776085
As	-0.977005	-2.358700	0.776085
As	0.977005	-2.358700	-0.776085
As	2.358700	-0.977005	0.776085

$[Ta@As_8]^{3–}$ E = –55.933 eV

Ta	-0.000000	0.000000	0.000000
As	-0.973679	2.350668	-0.782948
As	-2.350668	0.973679	0.782948
As	2.350668	0.973679	-0.782948
As	0.973679	2.350668	0.782948
As	-2.350668	-0.973679	-0.782948
As	-0.973679	-2.350668	0.782948
As	0.973679	-2.350668	-0.782948
As	2.350668	-0.973679	0.782948

$[Nb@As_8(ZnMes)_2]^{3–}$ E = –300.618 eV

Nb	0.319371	0.063711	-0.099043

As	-1.544486	1.856409	-0.986794
As	-1.051249	-0.173295	-2.449953
As	1.316756	0.453964	-2.608606
As	2.142662	-1.453716	-1.312302
As	0.714838	2.614619	0.092622
As	-0.216509	1.685622	2.246654
As	0.961467	-0.484121	2.439041
As	-0.999829	-1.721921	1.396739
Zn	-2.297758	0.571331	1.166918
Zn	-0.233210	-2.491327	-0.975080
C	-4.256970	0.490657	1.595394
C	-5.096444	-0.409425	0.891768
C	-4.859738	1.301785	2.588643
C	-6.470293	-0.490124	1.179786
C	-6.236372	1.205363	2.858390
C	-7.064024	0.312927	2.162748
H	-7.092768	-1.200189	0.624146
H	-6.674933	1.842534	3.634126
C	-0.743395	-4.349316	-1.511131
C	-1.809622	-4.681312	-2.382128
C	0.006613	-5.420665	-0.959156
C	-2.094347	-6.024787	-2.687466
C	-0.297940	-6.754393	-1.278031
C	-1.350699	-7.080617	-2.144702
H	-2.921530	-6.252924	-3.368526
H	0.302384	-7.559136	-0.839664
C	-8.545740	0.242627	2.441509
H	-8.952046	-0.750503	2.202443
H	-8.766564	0.463666	3.495815
H	-9.099377	0.976872	1.832882
C	-1.689661	-8.518230	-2.455961
H	-0.783051	-9.133387	-2.552968
H	-2.298495	-8.965496	-1.652886
H	-2.266021	-8.599677	-3.388206
C	-4.034503	2.292029	3.380270
H	-4.651092	2.846881	4.101888
H	-3.226851	1.785928	3.932731
H	-3.543322	3.019276	2.714538
C	-4.522834	-1.301014	-0.185195
H	-5.295752	-1.926485	-0.654230
H	-4.033094	-0.705981	-0.974419
H	-3.742054	-1.962794	0.226596
C	1.148196	-5.137768	-0.010138
H	0.798249	-4.546436	0.853287
H	1.609197	-6.063015	0.364034
H	1.929160	-4.532285	-0.501046
C	-2.671383	-3.602999	-2.996197
H	-2.061074	-2.871453	-3.550644
H	-3.420495	-4.023759	-3.682150
H	-3.201700	-3.030494	-2.219020

$[Ta@As_8(ZnMes)_2]^{3-}$ E = –300.023 eV

Ta	0.316923	0.055637	-0.088220
As	-1.546322	1.848076	-0.985370
As	-1.032076	-0.182655	-2.443069
As	1.341217	0.443770	-2.591624
As	2.156788	-1.454187	-1.256800
As	0.717939	2.590868	0.105064
As	-0.237911	1.694115	2.266859
As	0.949266	-0.471134	2.441745
As	-1.009292	-1.721650	1.387927
Zn	-2.305280	0.571899	1.163695
Zn	-0.221219	-2.500598	-0.966703
C	-4.264894	0.496673	1.590744
C	-5.105729	-0.412711	0.900311
C	-4.866630	1.319119	2.575558

C	-6.478260	-0.493551	1.195100
C	-6.241596	1.221712	2.853025
C	-7.069571	0.318031	2.172600
H	-7.101267	-1.211643	0.650525
H	-6.678369	1.866541	3.623476
C	-0.733104	-4.354465	-1.514848
C	-1.801090	-4.681262	-2.385871
C	0.015421	-5.429899	-0.968344
C	-2.088650	-6.023153	-2.696135
C	-0.291795	-6.761713	-1.292277
C	-1.346441	-7.082616	-2.158667
H	-2.917559	-6.247040	-3.376492
H	0.307416	-7.569281	-0.857576
C	-8.549042	0.244678	2.462219
H	-8.952877	-0.752623	2.236673
H	-8.763128	0.475923	3.515756
H	-9.110197	0.970356	1.850212
C	-1.688886	-8.518422	-2.474284
H	-0.783401	-9.133635	-2.582038
H	-2.290988	-8.969530	-1.668308
H	-2.273031	-8.594971	-3.402042
C	-4.041488	2.322393	3.350435
H	-4.655881	2.881700	4.070480
H	-3.226149	1.827216	3.901462
H	-3.559550	3.045321	2.673191
C	-4.535321	-1.314750	-0.169731
H	-5.308831	-1.948102	-0.627134
H	-4.053064	-0.726615	-0.968516
H	-3.749549	-1.969272	0.244262
C	1.158558	-5.153755	-0.019121
H	0.811178	-4.566752	0.848153
H	1.618556	-6.081699	0.349570
H	1.939661	-4.546754	-0.507871
C	-2.663250	-3.599561	-2.993202
H	-2.054190	-2.866854	-3.547446
H	-3.415368	-4.017040	-3.677877
H	-3.190011	-3.028978	-2.212149

$[Nb@As_8(ZnMes)_4]^{3-}$ E = –546.717 eV

Nb	0.000000	-0.000000	0.000000
As	-0.624146	2.406367	-1.184534
As	-1.172997	0.371932	-2.485383
As	1.172997	-0.371932	-2.485383
As	0.624146	-2.406367	-1.184534
As	2.406367	0.624146	1.184534
As	0.371932	1.172997	2.485383
As	-0.371932	-1.172997	2.485383
As	-2.406367	-0.624146	1.184534
Zn	-1.772788	1.927423	1.033057
Zn	1.927423	1.772788	-1.033057
Zn	1.772788	-1.927423	1.033057
Zn	-1.927423	-1.772788	-1.033057
C	3.210936	3.188560	-1.633465
C	3.171217	4.443644	-0.973574
C	4.165706	3.025609	-2.667385
C	4.040126	5.483406	-1.348539
C	5.022900	4.081235	-3.025937
C	4.977232	5.323382	-2.377579
H	3.982714	6.443363	-0.823621
H	5.747018	3.929448	-3.833886
C	-3.188560	3.210936	1.633465
C	-4.443644	3.171217	0.973574
C	-3.025609	4.165706	2.667385
C	-5.483406	4.040126	1.348539
C	-4.081235	5.022900	3.025937
C	-5.323382	4.977232	2.377579

H	-6.443363	3.982714	0.823621
H	-3.929448	5.747018	3.833886
C	-3.210936	-3.188560	-1.633465
C	-4.165706	-3.025609	-2.667385
C	-3.171217	-4.443644	-0.973574
C	-5.022900	-4.081235	-3.025937
C	-4.040126	-5.483406	-1.348539
C	-4.977232	-5.323382	-2.377579
H	-5.747018	-3.929448	-3.833886
H	-3.982714	-6.443363	-0.823621
C	3.188560	-3.210936	1.633465
C	3.025609	-4.165706	2.667385
C	4.443644	-3.171217	0.973574
C	4.081235	-5.022900	3.025937
C	5.483406	-4.040126	1.348539
C	5.323382	-4.977232	2.377579
H	3.929448	-5.747018	3.833886
H	6.443363	-3.982714	0.823621
C	-6.436559	5.924910	2.752320
H	-7.420498	5.507208	2.495896
H	-6.425467	6.151570	3.828470
H	-6.338841	6.884482	2.217818
C	6.436559	-5.924910	2.752320
H	6.425467	-6.151570	3.828470
H	6.338841	-6.884482	2.217818
H	7.420498	-5.507208	2.495896
C	-5.924910	-6.436559	-2.752320
H	-5.507208	-7.420498	-2.495896
H	-6.884482	-6.338841	-2.217818
H	-6.151570	-6.425467	-3.828470
C	5.924910	6.436559	-2.752320
H	6.884482	6.338841	-2.217818
H	6.151570	6.425467	-3.828470
H	5.507208	7.420498	-2.495896
C	1.712386	-4.286262	3.406643
H	0.886499	-4.508784	2.712121
H	1.746180	-5.080665	4.166064
H	1.446617	-3.340021	3.904221
C	-1.712386	4.286262	3.406643
H	-1.746180	5.080665	4.166064
H	-1.446617	3.340021	3.904221
H	-0.886499	4.508784	2.712121
C	-4.685674	2.188374	-0.148619
H	-5.680962	2.316877	-0.597579
H	-3.925549	2.301647	-0.939876
H	-4.598504	1.149102	0.211986
C	4.685674	-2.188374	-0.148619
H	4.598504	-1.149102	0.211986
H	5.680962	-2.316877	-0.597579
H	3.925549	-2.301647	-0.939876
C	2.188374	4.685674	0.148619
H	1.149102	4.598504	-0.211986
H	2.301647	3.925549	0.939876
H	2.316877	5.680962	0.597579
C	-2.188374	-4.685674	0.148619
H	-2.301647	-3.925549	0.939876
H	-2.316877	-5.680962	0.597579
H	-1.149102	-4.598504	-0.211986
C	-4.286262	-1.712386	-3.406643
H	-3.340021	-1.446617	-3.904221
H	-5.080665	-1.746180	-4.166064
H	-4.508784	-0.886499	-2.712121
C	4.286262	1.712386	-3.406643
H	4.508784	0.886499	-2.712121
H	3.340021	1.446617	-3.904221
H	5.080665	1.746180	-4.166064

[Ta@As$_8$(ZnMes)$_4$]$^{3-}$ E = –546.193 eV

Ta	-0.000000	-0.000000	0.000000
As	-0.622138	2.400083	-1.170760
As	-1.173212	0.371886	-2.490127
As	1.173212	-0.371886	-2.490127
As	0.622138	-2.400083	-1.170760
As	2.400083	0.622138	1.170760
As	0.371886	1.173212	2.490127
As	-0.371886	-1.173212	2.490127
As	-2.400083	-0.622138	1.170760
Zn	-1.769350	1.928454	1.042879
Zn	1.928454	1.769350	-1.042879
Zn	1.769350	-1.928454	1.042879
Zn	-1.928454	-1.769350	-1.042879
C	3.209123	3.183063	-1.652871
C	3.167787	4.442296	-1.000503
C	4.163457	3.016483	-2.686877
C	4.033375	5.481986	-1.383318
C	5.017557	4.071951	-3.053085
C	4.969400	5.318095	-2.412661
H	3.974378	6.445009	-0.864181
H	5.741189	3.916869	-3.860837
C	-3.183063	3.209123	1.652871
C	-4.442296	3.167787	1.000503
C	-3.016483	4.163457	2.686877
C	-5.481986	4.033375	1.383318
C	-4.071951	5.017557	3.053085
C	-5.318095	4.969400	2.412661
H	-6.445009	3.974378	0.864181
H	-3.916869	5.741189	3.860837
C	-3.209123	-3.183063	-1.652871
C	-4.163457	-3.016483	-2.686877
C	-3.167787	-4.442296	-1.000503
C	-5.017557	-4.071951	-3.053085
C	-4.033375	-5.481986	-1.383318
C	-4.969400	-5.318095	-2.412661
H	-5.741189	-3.916869	-3.860837
H	-3.974378	-6.445009	-0.864181
C	3.183063	-3.209123	1.652871
C	3.016483	-4.163457	2.686877
C	4.442296	-3.167787	1.000503
C	4.071951	-5.017557	3.053085
C	5.481986	-4.033375	1.383318
C	5.318095	-4.969400	2.412661
H	3.916869	-5.741189	3.860837
H	6.445009	-3.974378	0.864181
C	-6.431398	5.913555	2.795825
H	-7.415705	5.494596	2.542897
H	-6.415546	6.136902	3.872607
H	-6.338308	6.875005	2.263897
C	6.431398	-5.913555	2.795825
H	6.415546	-6.136902	3.872607
H	6.338308	-6.875005	2.263897
H	7.415705	-5.494596	2.542897
C	-5.913555	-6.431398	-2.795825
H	-5.494596	-7.415705	-2.542897
H	-6.875005	-6.338308	-2.263897
H	-6.136902	-6.415546	-3.872607
C	5.913555	6.431398	-2.795825
H	6.875005	6.338308	-2.263897
H	6.136902	6.415546	-3.872607
H	5.494596	7.415705	-2.542897
C	1.699225	-4.286600	3.418493
H	0.877546	-4.509500	2.719148
H	1.729919	-5.081774	4.177257

H	1.429301	-3.341362	3.915725
C	-1.699225	4.286600	3.418493
H	-1.729919	5.081774	4.177257
H	-1.429301	3.341362	3.915725
H	-0.877546	4.509500	2.719148
C	-4.689243	2.187129	-0.122692
H	-5.687049	2.315869	-0.565943
H	-3.933668	2.302957	-0.917847
H	-4.599269	1.146980	0.234796
C	4.689243	-2.187129	-0.122692
H	4.599269	-1.146980	0.234796
H	5.687049	-2.315869	-0.565943
H	3.933668	-2.302957	-0.917847
C	2.187129	4.689243	0.122692
H	1.146980	4.599269	-0.234796
H	2.302957	3.933668	0.917847
H	2.315869	5.687049	0.565943
C	-2.187129	-4.689243	0.122692
H	-2.302957	-3.933668	0.917847
H	-2.315869	-5.687049	0.565943
H	-1.146980	-4.599269	-0.234796
C	-4.286600	-1.699225	-3.418493
H	-3.341362	-1.429301	-3.915725
H	-5.081774	-1.729919	-4.177257
H	-4.509500	-0.877546	-2.719148
C	4.286600	1.699225	-3.418493
H	4.509500	0.877546	-2.719148
H	3.341362	1.429301	-3.915725
H	5.081774	1.729919	-4.177257

References

1 (a) J. J. Dunsford, E. R. Clark and M. J. Ingleson, *Angew. Chem., Int. Ed.,* 2015, **54**, 5688–5692; (b) B. Zhou, M. S. Denning, C. Jones and J. M. Goicoechea, *Dalton Trans.*, 2009, 1571 –1578.

2 (a) B. Kesanli, J. Fettinger, B. Scott and B. W. Eichhorn, *Inorg. Chem.,* 2004, **43**, 3840–3846; (b) W. Q. Zhang, H. W. T. Morgan, C. C. Shu, J. E. McGrady and Z. M. Sun, *Inorg Chem.*, 2022, **61**, 4421–4427.

3 O. V. Dolomanov, L. J. Bourhis, R. J. Gildea, J. A. K. Howard and H. Puschmann, *J. Appl. Cryst.,* 2009, **42**, 339–341.

4 (a) G. te Velde, F. M. Bickelhaupt, E. J. Baerends, C. Fonseca Guerra, S. J. A. van Gisbergen, J. G. Snijders and T. Ziegler, *J. Comput. Chem.*, 2001, **22**, 931–967; (b) AMS2021, SCM, Theoretical Chemistry, Vrije Universiteit, Amsterdam, The Netherlands, http://www.scm.com.

5 E. V. Lenthe and E.J. Baerends, *J. Comp. Chem.,* 2003, **24**, 1142–1156.

6 (a) J. P. Perdew, K. Burke and M. Ernzerhof, *Phys. Rev. Lett.,* 1996, **77**, 3865–3868; (b) J. P. Perdew, K. Burke and M. Ernzerhof, *Phys. Rev. Lett.,* 1997, **78**, 1396.

7 (a) E. V. Lenthe, E. J. Baerends and J. G. Snijders, *J. Chem. Phys.,* 1993, **99**, 4597-4610; (b) E. V. Lenthe, E. J. Baerends and J. G. Snijders, *J. Chem. Phys.,* 1994, **101**, 9783–9792; (c) E. V. Lenthe, A. E. Ehlers and E. J. Baerends, *J. Chem. Phys.*, 1999, **110**, 8943–8953.

8 C. C. Pye and T. Ziegler, *Theor. Chem. Acc.*, 1999, **101**, 396–408.
